# Challenges in the Diagnosis and Management of Graves' Disease in an Elderly Female With Antithyroid Drug Intolerance

**DOI:** 10.7759/cureus.94221

**Published:** 2025-10-09

**Authors:** Charlotte Soan, Charlotte Fernando, Kimberley Lambert, Meenakshi Parsad

**Affiliations:** 1 Diabetes and Endocrinology, Hampshire Hospitals NHS Foundation Trust, Winchester, GBR; 2 Diabetes and Endocrinology, Royal Hampshire County Hospital, Winchester, GBR

**Keywords:** anti-thyroid drugs, elderly female, graves´disease, graves' disease, lugol’s iodine, thyrotoxicity

## Abstract

Graves’ Disease (GD) is uncommon in the elderly. This report explores the case of an 87-year-old female diagnosed with Graves’ Disease following a non-specific decline and new-onset atrial fibrillation (AF). Incidental blood tests on admission revealed severe thyrotoxicosis with a thyroid-stimulating hormone (TSH) level of <0.0005mu/L and free thyroxine (fT4) level of 72 pmol/L, with positive TSH-receptor antibodies confirming Graves’ Disease. Initial management with carbimazole achieved some biochemical control but led to hepatotoxicity. Reduced dosing caused similar hepatic derangement. Propylthiouracil also did not adequately control thyroid function and caused mild liver function derangement. The patient re-presented with decompensated thyrotoxicosis, including hypotension, fast AF, and confusion. The endocrinology multidisciplinary team (MDT) decided the patient was unsuitable for definitive treatments; hence, Lugol’s iodine was commenced. Thyroid function gradually stabilised on a combination of Lugol’s iodine, propranolol, steroid and low-dose propylthiouracil.

This case was challenging due to anti-thyroid drug intolerance in conjunction with limited definitive management options, with our endocrinology team resorting to non-standard therapies to stabilise the patient. This case ultimately demonstrates the diagnostic and therapeutic challenges of managing Graves’ hyperthyroidism in the elderly.

## Introduction

Graves’ Disease (GD), the most common form of hyperthyroidism in iodine-sufficient countries [1], is an autoimmune disorder characterised by thyroid-stimulating hormone receptor (TSH) autoantibodies causing overproduction of thyroid hormones [2]. 

GD has a global prevalence of 2% in women and 0.5% in men [3], and typically presents between 20 and 50 years of age [4]. The incidence of thyroid disease in the elderly is slowly increasing, thought to be due to the progressively ageing population and an increased life expectancy [5]. However, the most common cause of hyperthyroidism in the elderly is thyrotoxic nodules as opposed to GD [4]. 

The typical features observed in younger individuals with GD include weight loss, anxiety, palpitations, diarrhoea and heat intolerance [3]. In contrast, elderly patients often present atypically, with a phenomenon known as 'apathetic thyrotoxicosis' [5], where more subtle features, such as lethargy, weight loss and cognitive decline, predominate [2,5]. These non-specific features overlap with other geriatric syndromes, such as non-specific infection, frailty, dementia, or vascular disease, which makes diagnosis more challenging and often delayed [5].

This report presents an atypical case of a new diagnosis of GD in an 87-year-old female, with no prior history of thyroid disease. She presented with a non-specific decline, including confusion, loss of appetite, weight loss and lethargy, along with new onset atrial fibrillation (AF). Initial investigations revealed significant thyrotoxicosis, with subsequent TSH-receptor antibodies found to be positive, consistent with GD.

Management of this patient was particularly challenging due to intolerance of both carbimazole and propylthiouracil (PTU), the two mainstay antithyroid drugs (ATDs), both of which resulted in liver function test (LFT) derangement. Due to her age, frailty, mild cognitive impairment and social dependency, she was also not a candidate for definitive management, including surgery or radioactive iodine therapies.

She was later admitted with decompensated thyrotoxicosis and cardiovascular instability, requiring cautious use of digoxin and beta-blockers. A short-term course of Lugol’s iodine was then trialled, alongside low-dose PTU, with close monitoring. Her thyroid function stabilised, and she was discharged to residential care.

This case is notable not only for the rarity of GD presenting in this age group and the diagnostic challenges associated with this, but also for the therapeutic difficulties encountered, especially when standard treatments were unsafe and poorly tolerated, and definitive management options were limited.

## Case presentation

An 87-year-old female, with a past medical history of deafness, glaucoma, polymyalgia rheumatica and osteoarthritis, was admitted to our district general hospital with a several-week history of fatigue, weight loss, poor appetite and confusion. This is in the background of a recent brief admission with dysphasia, which was felt to be a probable transient ischaemic attack (TIA).

On admission, she was found to be in new-onset AF, with a fast ventricular rate, mild hypotension, and intermittent confusion. These features, particularly new AF and confusion, were the first clinical indicators of a possible underlying metabolic cause. Importantly, there were no features of 'classical' hyperthyroidism such as tremor or heat intolerance. This is consistent with apathetic thyrotoxicosis observed in elderly populations. Similar presentations should prompt clinicians to consider thyroid function testing. 

She was initially treated with metoprolol and digoxin. A computerised tomography (CT) scan of the head revealed chronic lacunar infarcts with no acute pathology. She was commenced on telemetry, which showed possible, brief runs of ventricular tachycardia.

**Figure 1:**
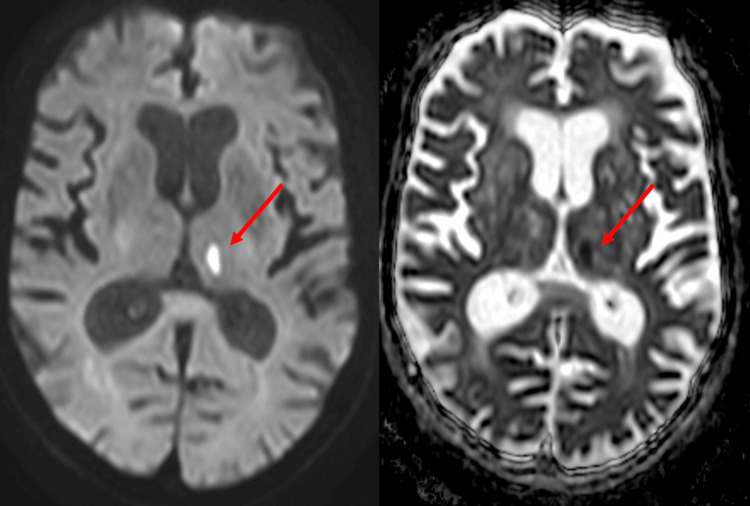
Magnetic resonance imaging (MRI) of the acute left thalamic infarct (indicated by red arrows), shown on diffusion-weighted imaging (DWI) (left), with an accompanying apparent diffusion coefficient (ADC) map (right)

Following advice from the stroke team, a magnetic resonance imaging (MRI) brain scan was performed (Figure 1), which demonstrated a new acute left thalamic infarct, with global atrophy and multiple cerebral microhaemorrhages, suggestive of underlying cerebral amyloid angiopathy.

Admission blood tests included thyroid function tests (TFTs), which revealed thyrotoxicosis, with a TSH level of <0.0005 mu/L, and a free thyroxine (fT4) level of 72 pmol/L (Table 1)*.* TSH receptor antibodies were subsequently found to be raised, supporting a new diagnosis of GD. Carbimazole 30 mg once a day was commenced, with repeat TFTs recommended within 6 weeks.

**Table 1:** Summary of patient laboratory results with corresponding dates and associated treatment adjustments made Thyroid-Stimulating Hormone (TSH), Free Thyroxine (FT4), Alanine Aminotransferase (ALT), Alkaline Phosphatase (ALP), Thyroid Function Tests (TFTs), Liver Function Tests (LFTs), Once Daily (OD), Thyrotropin Receptor Autoantibodies (TSHRAbs), Twice Daily (BD), Propylthiouracil (PTU), Three Times Daily (TDS), Multidisciplinary Team (MDT), Radioactive Iodine Therapy (RAI)

Date	Test	Value	Reference Range	Action taken as a result
03/08/2024	TSH	<0.005 mu/L	0.27-4.2 mu/L	Discussed with the endocrinology team - suggested to commence carbimazole 30 mg OD for 6 weeks. Then, repeat TFTs. Advised to send TSHRAbs, safety netting advice regarding agranulocytosis provided. Discharged to an interim care facility.
	FT4	72 pmol/L	11-22 pmol/L	
	ALT	26 U/L	0-35 U/L	
	ALP	102 U/L	30-130 U/L	
06/08/2024	TSH-Receptor Antibodies	10.4 IU/L	0-0.4 IU/L	Consistent with Graves’ Disease. No change to the above plan.
07/09/2024	TSH	0.108 mu/L	0.27-4.2 mu/L	Carbimazole suspended 11/09/24. Advised to recheck LFTs in 2 weeks and restart if LFT derangement improves.
	FT4	9.7 pmol/L	11-22 pmol/L	
	ALT	235 U/L	0-35 U/L	
	ALP	608 U/L	30-130 U/L	
02/10/2024	TSH	<0.005 mu/L	0.27-4.2 mu/L	Advised to restart carbimazole at a lower dose (20 mg OD).
	FT4	69 pmol/L	11-22 pmol/L	
	ALT	22 U/L	0-35 U/L	
	ALP	135 U/L	30-130 U/L	
09/10/2024	ALT	150 U/L	0-35 U/L	Stop carbimazole. Wait 1 week, then commence propylthiouracil 50 mg BD.
	ALP	354 U/L	30-130 U/L	
22/10/2024	TSH	<0.005 mu/L	0.27-4.2 mu/L	Admitted to resus from the endocrinology outpatient clinic. Commenced prednisolone 20 mg OD for 1 week, digoxin 62.5 mcg, propranolol 40 mg TDS, and commenced 50 mg PTU OD (conscious decision in view of deranged LFTs on carbimazole).
	FT4	62 pmol/L	11-22 pmol/L	
	ALT	27 U/L	0-35 U/L	
	ALP	148 U/L	30-130 U/L	
24/10/2024	TSH	<0.005 mu/L	0.27-4.2 mu/L	Discussed in endocrinology MDT: LFTs mildly deranged since commencing PTU. Not a candidate for carbimazole (significant LFT derangement). Not a candidate for surgery or RAI. Decision to give Lugol’s iodine solution 0.3mls TDS for 10 days. Continue PTU at 50 mg BD.
	FT4	44 pmol/L	11-22 pmol/L	
	ALT	99 U/L	0-35 U/L	
	ALP	138 U/L	30-130 U/L	
30/10/2024	ALT	41 U/L	0-35 U/L	PTU had been uptitrated to 100 mg BD.
	ALP	129 U/L	30-130 U/L	
02/11/2024	TSH	<0.005 mu/L	0.27-4.2 mu/L	Patient discharged to residential facility out of area.
	FT4	19.7 pmol/L	11-22 pmol/L	
	ALT	25 U/L	0-35 U/L	
	ALP	114 U/L	30-130 U/L	

Thyroid sonography was not performed in this case, as GD was confirmed serologically. Given the patient's frailty and the absence of a palpable thyroid nodule, further imaging was not clinically indicated.

The patient’s thyroid function was well-controlled on carbimazole, with a repeat TSH of 0.108 mu/L and fT4 of 9.7 pmol/L/L. Clinically, the patient's confusion appeared to improve slightly, in parallel with the reduction in fT4 levels, consistent with some resolution of thyrotoxicosis. However, shortly after initiation, the patients’ LFTs started to become deranged, with alanine aminotransferase (ALT) rising to 286 U/L, and alkaline phosphatase (ALP) rising to 602 U/L (Table 1). Carbimazole was suspended, and the patient was discharged to an interim care facility, with the plan to repeat LFTs in two weeks, and to consider restarting carbimazole if LFTs normalised.

Repeat LFTs showed moderate improvement, and the endocrinology team advised restarting carbimazole at a lower dose of 20 mg once a day. Unfortunately, this was not tolerated by the patient, and blood tests showed continued derangement in ALT to 150 U/L and ALP to 354 U/L (Table 1). In view of carbimazole-induced hepatotoxicity, treatment was discontinued.

On endocrinology advice, PTU 50 mg twice daily was suggested as a second-line management option.

The patient attended endocrinology outpatient follow-up and was found to be hypotensive and tachycardic, with acute confusion. She was transferred directly from the clinic to the emergency department resuscitation area and again commenced on telemetry. An electrocardiogram (ECG) confirmed AF with a fast ventricular rate, sometimes exceeding 160 bpm. She was treated with propranolol, intravenous metoprolol, hydrocortisone and fluid resuscitation. A diagnosis of decompensated thyrotoxicosis was made.

Once stabilised, she was commenced on propranolol 40 mg three times daily, which was later up-titrated. PTU was cautiously initiated, given previous hepatotoxicity with carbimazole.

The patient’s case was discussed in the endocrine multidisciplinary team (MDT), and it was decided that she was not a candidate for thyroidectomy due to her frailty and cognitive fluctuation and was not suitable for radioactive iodine due to significant care needs.

Two days later, thyroid hormone levels remained markedly elevated with an fT4 of 62 pmol/L and T3 of 24.6 pmol/L, and liver enzymes began to rise again despite the low-dose PTU (Table 1). In view of limited pharmacological options, Lugol’s iodine was commenced at 0.3 mL three times a day to inhibit thyroid hormone synthesis and release. Corticosteroids were discontinued once stabilised from acute thyrotoxicosis.

Despite the above therapy, the patient remained tachycardic and hypotensive over the following days, with a National Early Warning Score (NEWS) score of 8. Lugol’s iodine was up-titrated to 50 mg twice daily. Propranolol was also increased. Liver function gradually stabilised, and PTU was cautiously up-titrated to 100 mg twice daily. Lugol’s iodine was subsequently discontinued.

Repeat bloods demonstrated improving thyroid function, T3 having fallen to 3.7 pmol/L and T4 to 19.7 pmol/L, although TSH remained suppressed (<0.0005 mu/L) (Table 1). The patient was medically optimised and discharged to a care home, with plans for community monitoring and outpatient endocrine follow-up.

## Discussion

GD in the elderly is relatively uncommon, given that the peak incidence of presentation is between 20 and 50 years [4]. In elderly patients, the typical features of GD, such as palpitations, tremor, diarrhoea and heat intolerance [3], may be absent, masked by comorbidities, or attributed to ageing. Around 20-40% of elderly hyperthyroid patients present with ‘apathetic thyrotoxicosis’ -- a phenomenon well-documented within the literature [2,4], whereby patients present with subtle symptoms such as lethargy, depression, weight loss and reduced appetite. This relative non-specificity of symptoms can lead to diagnostic delays, and symptoms can instead be attributed to other diagnoses such as infections, delirium or malignancy [6]. In this patient’s case, her presentation was considered in the context of prior stroke, and only incidentally was her thyroid function tested on admission. This highlights the importance of clinicians having a high index of suspicion for thyroid dysfunction and a low threshold for testing thyroid function as part of the initial workup for elderly patients admitted with non-specific symptoms.

Hyperthyroidism is associated with significant cardiovascular risk, including AF, acute myocardial infarction, heart failure, venous thromboembolism and stroke [7]. AF is the most common cardiac manifestation observed in patients with hyperthyroidism [5]. Indeed, AF may be the only presenting feature in up to 40% of elderly patients with hyperthyroidism [2]. In this patient’s case, AF may have been the first clinical indicator of hyperthyroidism during her first admission for probable transient ischaemic attack (TIA), given that subsequent imaging revealed embolic-type infarcts. Retrospectively, it may be plausible that she had hyperthyroidism for some time, and that her AF had been paroxysmal or asymptomatic. This further supports routine TFT testing in new presentations of AF in the elderly.

Initial management of GD includes antithyroid drugs (ATDs), such as carbimazole and propylthiouracil (PTU), alongside symptomatic management with beta blockers for relief of adrenergic features of hyperthyroidism [8,9]. ATDs are generally well-tolerated, with the most common side effects being rash, urticaria and arthralgia [2]. It is also widely accepted that all ATDs can affect the liver in some way, given its important role in metabolising thyroid hormones [10]. ATD-related hepatotoxicity is rare (affecting 0.1-0.2% of treated patients) but can manifest in a spectrum of presentations, from mild LFT derangement to acute liver failure, with some reported cases even requiring transplant [11]. Risk of LFT derangement is greater in elderly populations on carbimazole, while, in contrast, PTU-associated hepatotoxicity is more common in paediatric patients [11].

More definitive therapies in the management of GD are radioactive iodine ablation therapy or surgical thyroidectomy, which both aim to reduce the volume of active thyroid tissue [2,3]. Guidelines from the European Thyroid Association (ETA) recommend definitive therapies as the first-line management, particularly in older patients who present with AF or other cardiac complications [2]. However, our patient was deemed unsuitable for either of the definitive treatments due to frailty, cognitive impairment, and her increased care needs (necessary to support her activities of daily living).

Our patient was therefore commenced on carbimazole, but unfortunately, despite achieving some biochemical response, the patient developed hepatotoxicity and, hence, this had to be discontinued. A subsequent trial of PTU also led to deranged LFTs, necessitating its withdrawal.

During her final admission, the patient exhibited signs of acute cardiovascular compromise (hypotension, tachycardia), raising concerns for thyroid storm. Initial rate control was challenging due to hypotension, requiring cautious use of digoxin, and later introduction of beta blockers once she had stabilised. Steroids were also essential in managing her acute presentation.

As a result, the endocrinology MDT decided to commence Lugol’s solution. This is an older treatment modality, which is no longer widely used or recommended within modern literature. By virtue of the Wolff-Chaikoff phenomenon, the effect of Lugol’s solution may be rapid (excess iodide causing transient inhibition of thyroid synthesis) [11]. However, this effect is often short-lived; hence, it does not provide a reliable long-term control of thyroid function [12]. This, in combination with the advancement of other pharmacological agents with more predictable side effect profiles [11], means that Lugol’s solution has gone out of favour. It can still be used in refractory cases, for urgent control in thyroid storm, pre-operatively, or in cases where ATDs are contraindicated or not tolerated [12].

Ultimately, Lugol’s solution was essential in the management of our patient’s disease, and her thyroid function stabilised following the completion of this therapy.

## Conclusions

This case ultimately highlights the diagnostic and therapeutic complexities of managing Graves’ Disease in the elderly. Atypical presentations, co-morbidities and management options being limited by frailty all complicate the care of this population. Our patients’ presentation with new atrial fibrillation and embolic events highlights the subtlety of thyroid disease in the elderly. Furthermore, our patients’ intolerance of first-line antithyroid drugs, as well as ineligibility for definitive therapies, necessitated the use of non-standard interventions. This highlights the importance of an individualised approach, combined with clinical flexibility. Clinicians should maintain a high index of suspicion for thyroid dysfunction in older adults. Individualised care and multidisciplinary strategies are essential in this population.
